# Autophagy-Associated lncRNAs: Promising Targets for Neurological Disease Diagnosis and Therapy

**DOI:** 10.1155/2020/8881687

**Published:** 2020-09-22

**Authors:** Xiangming Xu, Lili Cui, Wangtao Zhong, Yujie Cai

**Affiliations:** ^1^Guangdong Key Laboratory of Aged-Related Cardiac and Cerebral Diseases, Affiliated Hospital of Guangdong Medical University, Zhanjiang, China; ^2^Department of Neurology, Affiliated Hospital of Guangdong Medical University, Zhanjiang, China

## Abstract

Neurological diseases are a major threat to global public health and prosperity. The number of patients with neurological diseases is increasing due to the population aging and increasing life expectancy. Autophagy is one of the crucial mechanisms to maintain nerve cellular homeostasis. Numerous studies have demonstrated that autophagy plays a dual role in neurological diseases. Long noncoding RNAs (lncRNAs) are a vital class of noncoding RNAs with a length of more than 200 nucleotides and cannot encode proteins themselves but are expressed in most neurological diseases. An early phase, emerging knowledge has revealed that long noncoding RNAs (lncRNAs) are crucial in autophagy regulation. Furthermore, autophagy-associated lncRNAs can promote the development of neurological diseases or slow their progression. In this review, we introduce a general overview of lncRNA functional mechanisms and summarizes the recent progress of lncRNAs on autophagy regulation in neurological diseases to reveal possible novel therapeutic targets or useful biomarkers.

## 1. Introduction

Neurological diseases are important causes of human disability and death worldwide. According to the pathology, they can be divided into several groups, including cerebrovasculardiseases, neurodegenerative diseases, demyelinating diseases, infectious diseases, brain tumors, epilepsy, and headache. Most neurological diseases occur after the developmental maturation of CNS [1]. Autophagy is an important catabolic process during which the unwanted cytoplasmic components such as damaged organelles and protein aggregates are sequestered and engulfed by double-membrane vesicles called autophagosomes, and then autophagosomes fuse with lysosomes to form autolysosomes where the cargos are degraded and recycled [2–4]. Autophagy can be divided into basal autophagy and induced autophagy [5]. Several studies have demonstrated that basal autophagy plays a housekeeping role in eukaryotes through degrading useless and dysfunctional proteins and organelles to maintain cellular homeostasis and promote cell growth and development [3, 6]. Compared with basal autophagy, the degree of induced autophagy is significantly increased, which is a defensive response of the body to external stimuli and may cause autophagic cell death [5]. Neuronal autophagy plays an essential role in synaptic plasticity, oligodendrocyte development, anti-inflammatory function in glial cells, and myelination process [7, 8]. As postmitotic cells, nerve cells are unable to dispose toxic or misfolded proteins through cell division. Therefore, proper autophagy is crucial for nerve cells to remove harmful cellular components; however, insufficient activation of autophagy or pathological stress induced-autophagy will lead to the accumulation of those harmful constituents and eventually causes neuronal dysfunction, which is associated with neurological diseases such as Alzheimer's disease (AD), Parkinson's disease (PD), ischemic stroke (IS), and glioma [9].

Recently, noncoding RNA, such as long noncoding RNA (lncRNA), have been verified to regulate cell autophagy by several mechanisms and further contribute to many of the characteristics of disease phenotypes [10]: (1) lncRNA can sponge some miRNAs which directly target autophagy-related proteins to regulate autophagy. For example, lncRNA APF regulates miR-188-3p, thereby affecting the expression of ATG7 which is an autophagy factor [11]. FLJ11812 can regulate the level of the miR-4459 target ATG13 (autophagy-related 13) and then promote autophagy [12]. Similarly, lncRNA TGFB2-OT1 has been found to regulate the expression of autophagy-related proteins CERS1, NAT8L, and LARP1 by binding to miR-3960, miR-4488, and miR-4459 [13]. (2) lncRNA can also target autophagy-related signaling pathways, such as lncRNA H19 and lncRNA HOTAIR. In this review, we will discuss not only the current knowledge about the relationship between autophagy and neurological diseases but also the possible biological functions of lncRNA in regulating autophagy and summarize some specific studies that have provided novel insights into the underlying mechanism of the lncRNA-autophagy axis for neurological disease pathogenesis and therapeutic intervention.

## 2. lncRNA Characteristics, Classification, and Functional Mechanisms

**Figure 1:**
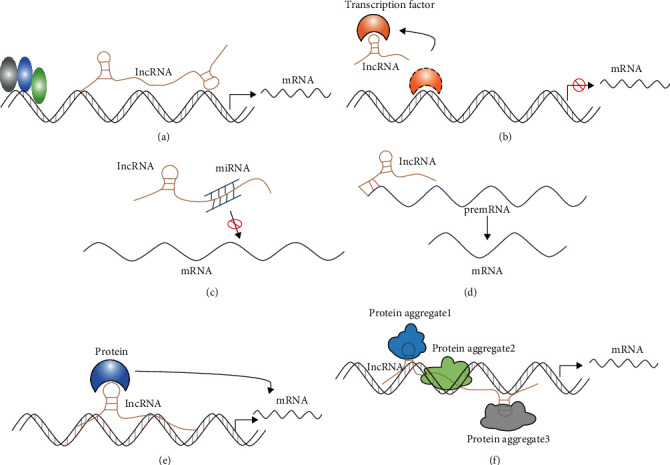
The modes of action of lncRNA. (a) lncRNAs function as signals to directly regulate the transcription of the downstream gene. (b) lncRNAs function as decoys to regulate the binding of proteins to DNA or other proteins. (c) lncRNAs interact with miRNA as miRNA sponges. (d) lncRNAs regulate alternative splicing. (e) lncRNAs serve as guide RNAs to impart specificity at genomic positions through either RNA-DNA or RNA-protein-DNA interactions. (f) lncRNAs serve as scaffolds to allow the formation of larger RNA-protein complexes.

Long noncoding RNAs (lncRNAs) are a vital class of noncoding RNAs with a length of more than 200 nucleotides. Surprisingly, although lncRNAs cannot encode proteins themselves, they are known to be 5′ capped, 3′ polyadenylated, and spliced similar to mRNAs. According to their location in the genome relative to nearby protein-coding genes, lncRNAs are generally divided into major groups: sense lncRNAs, antisense lncRNAs, bidirectional lncRNAs, enhancer lncRNAs, intronic lncRNAs, and intergenic lncRNAs [14–16]. They are widespread in most eukaryotic transcriptome and constitute a significant fraction of mammalian genomes, which are involved in cell growth, proliferation, differentiation, apoptosis, metabolism, and other biological processes [17, 18]. With the rapid development of high-throughput sequencing and gene chip technology, a large number of studies have indicated that lncRNA participates in the pathophysiology of various diseases, such as cancer, aging, cardiovascular and cerebrovascular diseases, and neurodegenerative disorders; however, its underlying molecular mechanism is still not well clarified [19–21]. The latest study showed that lncRNA functions generally by five modes of action: signals, decoys, sponges, guides, and scaffold [22, 23] (Figure 1). (1) In response to cell signaling or other stimuli from extracellular environments, lncRNAs that serve as signals will be transcribed and directly regulate the transcription of the downstream gene (Figure 1(a)). This process is directly regulated by lncRNA and does not involve protein translation, so it can respond to external stimuli quickly [24]. (2) lncRNAs can act as a decoy to bind to transcription factors or transcriptional regulating factors and then block its molecular action or other signaling components that regulate the transcription of downstream genes [23] (Figure 1(b)). (3) Interestingly, lncRNAs often function as competitive endogenous RNA (ceRNAs), namely, miRNA sponges or antagonists [25]. More specifically, lncRNAs can bind to miRNA with base pair sequence complementarity, which will repress the binding of miRNA to the 3′ untranslated regions (UTRs) of its target mRNA, protecting mRNA from degrading and consequently regulating protein translation [26, 27] (Figure 1(c)). (4) Besides, lncRNAs are also involved in posttranscriptional modification and alternative splicing of mRNA [28] (Figure 1(d)). (5) lncRNAs which serve as guide RNAs are classified into two categories *cis*-acting and *trans*-acting. These lncRNAs can combine with proteins such as transcription factors and transcriptional regulating factors and direct these RNA-protein complexes to the specific DNA sites, contributing to regulating transcription precisely [23] (Figure 1(e)).

(6) lncRNAs that act as a scaffold are like a central platform, and multiple relevant transcription factors can be bound to these lncRNAs to regulate the activity of transcription factors. Besides, multiple signaling pathways are activated or inhibited, and their downstream effector molecules can be bound to the platform to achieve information intersection and integration between different signaling pathways [29–31] (Figure 1(f)). It is worth noting that the above-mentioned modes of action of lncRNA do not exist independently, but are interrelated and interact with each other. Numerous studies have revealed that lncRNAs are highly expressed in a mammalian brain tissue and play a significant role in regulating protein-coding gene expression through the epigenetic, transcriptional, and posttranscriptional levels and are involved in multiple signaling pathways of various neurological diseases, which has a very broad clinical application prospect [32–34].

## 3. lncRNA as Regulators of Autophagy in Neurological Diseases

Autophagy is one of the crucial mechanisms to maintain nerve cellular homeostasis; however, numerous researches have shown that autophagy is a double-edged sword that can either protect cells against apoptosis or promote autophagic cell death. The activation of autophagy has been documented in various diseases such as cancer, AD, PD, and vascular diseases [35, 36]. Similar to other signaling pathways, autophagy is regulated by several factors, including transcription factors and noncoding RNAs such as lncRNAs and miRNAs. There is growing evidence that noncoding RNAs regulate diverse pathophysiological processes in vivo and in vitro, from cell proliferation to aging, are both closely associated with autophagy. Besides this, the deregulation of lncRNAs will contribute to numerous neurological diseases [37]. In this section, we will introduce some recent findings that shed light on the role of lncRNA-mediated autophagy in some common neurological diseases (Table 1).

### 3.1. lncRNA-Mediated Autophagy in Alzheimer's Disease

Alzheimer's disease (AD) is one of the most common age-related neurodegenerative diseases and causes progressive memory impairment and cognitive dysfunction, accounting for 70% of cases of dementia [38]. However, the etiology and pathogenesis of AD remain unclear. At present, most scholars believe that the extracellular disposition of *β*-amyloid (A*β*) and the intraneuronal accumulation of tau protein are two major neuropathological features of AD. Studies have shown that autophagy inducers seem to prevent the accumulation of *β*-amyloid plaques and neurofibrillary tangles by degrading these aggregates in the early stages of AD, while the promotion of autophagy could aggravate the impaired autophagosome-lysosomal fusion and lysosomal dysfunction the late stages of AD [39]. Despite great advances in diagnostic and therapeutic drugs of AD in recent years, there are no effective treatments to prevent or reverse AD progression. Hence, understanding the regulatory mechanisms underlying autophagy will be the focus of treatment for AD. Recent studies found that lncRNAs were differentially expressed in the blood and CSF from patients with AD compared to the healthy elderly and played a key role in AD pathogenesis [40]. Nevertheless, there are few reports about the role and molecular mechanism of lncRNAs on autophagy in AD; here, we introduce lncRNAs involved in autophagy regulation in the AD, which contributes to AD pathogenesis and provides new diagnostic biomarkers or therapeutic targets for AD.

#### 3.1.1. lncRNA 17A

lncRNA 17A is transcribed from the antisense strand of the GABABR2 gene and expressed in the human brain. Massone et al. suggested that the expression of lncRNA 17A was upregulated in the brain tissue of patients with AD and regulated A*β* secretion [41]. Wang et al. found that compared to lncRNA 17A overexpressed cells, lncRNA 17A knockdown increased the expression levels of LC3-II which is a hallmark of autophagosome formation. At the same time, the level of A*β*42 was diminished in shRNA-17A-transfected SH-SY5Y cells. A*β*42 is considered the main component of senile plaques and the primary mechanistic factor in AD pathology. Moreover, GABABR2 was found to be upregulated when lncRNA 17A was overexpressed and downregulated when lncRNA was knocked down. Therefore, the researchers speculated that depletion of lncRNA 17A promoted autophagy by regulating alternative splicing of GABABR2 and thus alleviated the accumulation of protein aggregates, which effectively suppress the progression of AD [42]. However, the effects of lncRNA 17A are needed to be examined in vivo.

#### 3.1.2. lncRNA NEAT1

lncRNA NEAT1 has been reported to be a vital transcriptional regulator in cancer cell growth [43]. NEAT1 was well documented to be upregulated in the brain tissue from patients with AD, and its role in the pathophysiology of AD has received much attention in recent years [44, 45]. lncRNA NEAT1 is significantly upregulated in old APP/PS1 mice (over 6 months old) in a time-dependent manner but not in younger littermates, and the levels of A*β* showed a similar expression pattern. A further study found that overexpression of lncRNA NEAT1 could improve the interaction of PINK1 and NEDD4L and facilitate PINK1 ubiquitination. Besides, protein levels of the autophagy adaptors such as P62, OPTN, and LC3 were decreased at the same time A*β* was increased. Furthermore, lncRNA NEAT1 knockdown can reverse the above-mentioned changes and ameliorates cognitive impairments in AD mice. These studies suggested that lncRNA NEAT1 can promote NEDD4L-mediated PINK1 ubiquitination and degradation and thus inhibit PINK1-dependent mitophagy, which finally escalates A*β* accumulation and cognitive decline [46]. It is well known that PINK1 together with Parkin can promote the recruitment of autophagy receptor OPTN and activate ubiquitin and autophagic proteins, contributing to the information of autophagosome [47]. From what has been discussed above, lncRNA NEAT1 aggravated A*β*-induced neuronal damage via promoting the ubiquitination and degradation of PINK1. Hence, lncRNA NEAT1 may be a useful biomarker for AD.

### 3.2. lncRNA-Mediated Autophagy in Parkinson's Disease

PD is the second most common neurodegenerative disorder after AD in aging individuals, the main characteristics of which are the progressive loss of dopaminergic neurons in the substantia nigra pars compacta and the formation of Lewy bodies within the cytoplasm, leading to motor dysregulation [48, 49]. Although the pathogenesis remains not fully understood, PD is widely assumed to be associated with both genetic and environmental factors [35]. Most PD cases are sporadic with an unclear etiology, but about 5% are familiar caused by genic mutations, including SNCA, LRRK2, PRKN, PINK1, MAPT, GBA, and PARK2 [50]. Mutations in these genes can cause the formation of cytotoxic aggregates, impaired actin remodeling, dysregulation of autophagy, and enhancement of proapoptotic signaling pathways and eventually lead to neuron degeneration. Recently, growing evidences indicated that lncRNA takes part in the pathogenesis of PD. In the following paragraphs, we focus on the role of lncRNA-mediated autophagy in PD.

#### 3.2.1. lncRNA NEAT1

Similar to its role in AD, lncRNA NEAT1 has also been proved to play key roles in PD pathophysiology. MPTP can significantly promote the expression level of lncRNA NEAT1, PINK1, and LC3-II in vitro and in vivo models of PD, which also seems to increase with dose and time within a certain range [51, 52]. Upregulated NEAT1 expression can further increase PINK1 expression level by inhibiting CHX-induced PINK1 protein degradation, which occurs both in impaired and in intact mitochondria. Moreover, the downregulation of NEAT1 largely reversed the effect of MPP+ on SH-SY5Y cells, including decreased LC3-II and PINK1 protein levels. The accumulated PINK1 directly interacts with LC3-II and increases the accumulation of LC3-II in mitochondria, leading to abnormal mitochondrial autophagy [51]. Interestingly, the NEAT1/PINK1/ LC3-II axis is involved not only in the degradation of damaged mitochondria in PD models but also in the abnormal elimination of the healthy mitochondria leading to reduced ATP generation thereby causing neurodegeneration [53]. The above results indicated that lncRNA NEAT1 could induce abnormal autophagy by stabilizing PINK1 which was an LC3-II upstream regulatory factor and played a role in the pathogenesis of PD.

#### 3.2.2. lncRNA SNHG1

It has been reported that the expression level of lncRNA SNHG1 was significantly upregulated in postmortem midbrain samples from PD patients compared to healthy people [54]. Besides, lncRNA SNHG1 also has been demonstrated to promote *α*-synuclein aggregation and enhance neurotoxicity resulting in dopaminergic neuronal loss [55]. Recent studies have found lncRNA SNHG1 gradually upregulated in cell and animal models in PD. Furthermore, silencing lncRNA SNHC1 could remarkably increase the expression of miR-221/222 and LC3-II and prevent MPP+-induced cell death. Further investigations indicated that lncRNA SNHG1 acted as a ceRNA and prevented miR-221/222 from interacting with target P27 mRNA which is a key regulatory factor for the phosphorylation of mTOR and cell death. The decreased expression of P27 can inhibit the mTOR pathway, thus promoting neural autophagy and alleviating MPP+-mediated cell injury [56]. Experiment result shows that downregulated lncRNA SNHG1 inhibits the mTOR pathway and initiates autophagy through sponging miR-221/222, which can reduce the death of dopaminergic neurons in PD patients.

#### 3.2.3. lncRNA HOTAIR

Several studies have indicated that lncRNA HOTAIR obviously increased in vivo and vitro models of PD [1, 57]. As previously mentioned, mutations of LRRK2 are associated with familial PD and they can enhance autophagy by activating the ERK/MAPK pathway [58]. Overexpression of lncRNA HOTAIR could specifically improve LRRK2 mRNA stability and upregulate its expression, which promoted autophagy in PD models. On the contrary, silencing HOTAIR would rescue these alterations and increase cell viability [57]. These results strongly indicated that lncRNA HOTAIR can activate the ERK/MAPK pathway through improving the stability of LRRK2, which can inhibit autophagy and ultimately lead to PD. Recently, Lin et al. found that lncRNA HOTAIR was upregulated in MPP+-treated SH-SY5Y cell lines and contribute to PD while downregulating HOTAIR would increase the number of TH-positive cells and decrease the number of *α*-synuclein-positive cells. Similar results were also observed in in vivo experiments. A further study showed that miR-126-5p negatively regulated the expressions of RAB3IP which have been demonstrated to inhibit autophagy in mammalian cells. lncRNA HOTAIR could prevent miR-126-5P from interacting with RAB3IP by sponging miR-126-5P, which would inhibit autophagy and eventually caused the accumulation of *α*-synuclein in dopaminergic neurons [59]. Overall, the lncRNA HOTAIR/miR-126-5p/RAB3IP axis has been proved to be related to autophagy in PD, and its dysregulation was considered a therapeutic target for PD.

#### 3.2.4. lncRNA HAGLROS

A recent study showed that lncRNA HAGLROS was upregulated and miR-100 was downregulated in MPTP-induced mice and MPP+-treated SH-SY5Y cells. Knockdown of lncRNA HAGLROS could increase the expression level of miR-100 and alleviated MPTP-induced autophagy. A further study found that ATG 10 which participated in the formation of autophagosomes and initiated autophagy was a direct target of miR-100 [60]. lncRNA HAGROS could prevent miR-100 from interacting with ATG10 by serving as a sponge of miR-100 and thus promote autophagy, leading to aggravating MPP+-induced cell injury. In addition, the PI3K/AKT/mTOR pathway was found to be inactivated in MPP+-intoxicated SH-SY5Y cell, which led to the decrease in the number of dopamine-positive cells and aggravated cell damage; meanwhile, lncRNA HAGLROS silencing could dramatically increase the phosphorylation levels of the PI3K/AKT/mTOR pathway and thus promote autophagy, contributing to protecting neurons from MPTP-induced damage [61]. Together, lncRNA HAGLROS regulates autophagy through regulating miR-100/ATG10 axis and PI3K/AKT/mTOR, which provide a potential therapeutic strategy for PD and need further evidence.

### 3.3. lncRNA-Mediated Autophagy in Ischemic Stroke

IS is a generic term for blood flow interruption and cerebral tissue necrosis caused by a thrombotic or embolic blockage of a cerebral artery, accounting for approximately 85% of all strokes [62, 63]. After brain ischemia, nerve cell membrane potential and cellular ion homeostasis are disrupted leading to a series of deleterious events in the brain such as excitotoxicity, oxidative stress, autophagy, and inflammation. These events affect each other, forming a positive feedback loop and causing ischemic cascading effects, which causes irreversible neuronal injury characterized by neuronal apoptosis or death in the core area, brain edema, and blood-brain barrier dysfunction [64, 65]. The current research indicated that autophagy was activated after cerebral ischemia; at this time, inhibition of autophagy plays a protective role whereas excessive activation of autophagy aggravates the injury. However, the inhibition of basal autophagy before ischemic stroke can aggravate subsequent cerebral ischemic injury [66, 67]. Therefore, autophagy induction may be a potential therapeutic target for IS [68]. We will review some regulatory lncRNAs on autophagy in IS in this section.

#### 3.3.1. lncRNA H19

lncRNA H19, a maternally imprinted gene, generally declines after birth, whereas it increases in pathological situations such as cancer, oxidative stress, or hypoxia, which is important for early embryonic development [69]. The expression levels of lncRNA H19 are dramatically increased in the peripheral blood of ischemic patients and brain tissue, plasma, and white blood cells of mice with transient cerebral ischemia. Wang et al. found that compared to the control group, the human neuroblastoma cell line SH-SY5Y subjected to oxygen-glucose deprivation/reoxygenation (OGD/R) significantly induced the expression of lncRNA H19, and at the same time, the ratio of LC3II/I and beclin1 was increased, while P62 was decreased. Knocking out lncRNA h19 has been reported to reverse these changes. Further studies have shown that the overexpression of lncRNA H19 can activate autophagy by inhibiting DUSP5, a mitogen-activated protein kinase phosphatase, and thus activating ERK1/2 which is related to autophagy initiation [70, 71]. Taken together, lncRNA H19 can promote autophagy through regulating the DUSP5-ERK1/2 axis, contributing to cerebral ischemia-reperfusion injury. However, these results still need to be further verified by in vivo experiments.

#### 3.3.2. lncRNA MALAT1

lncRNA MALAT1 was considered to be one of the most significantly upregulated lncRNAs in both in vivo and in vitro models of IS, accompanied by the upregulation of LC3-II and Beclin1 [72, 73]. Further studies by Guo et al. showed that lncRNA MALAT1 decreases Beclin1 expression by acting as a sponge for miR-30a and thus promotes Beclin1-dependent autophagy, leading to neuronal cell death after IS. Moreover, lncRNA MALAT1 silencing can alleviate ischemic brain injury by inhibiting autophagy. This suggests that the MALAT1/miR-30a/beclin1 (lncRNA/miRNA/mRNA) regulatory network may exist in ischemic stroke [73]. Interestingly, lncRNA MALAT1 and autophagy have a protective effect on BMECs during IS. MALAT1 functions as a ceRNA for miR-26b, which can promote ULK2 expression. ULK2 is a downstream target in the mTOR signaling pathway and is associated with autophagosome formation, suggesting that MALAT1 protected BMECs from ischemia-reperfusion injury by promoting autophagy [74]. Another study by Wang et al. reported that the in vitro BMEC model, lncRNA MALAT1, also serves as ceRNA and prevents miR-200c-3p from binding to Sirt1 which has been reported to stimulate the expression and deacetylation of autophagy-related genes, suggesting that lncRNA MALAT1 can activate autophagy and promote the survival of OGD/R-treated BMECs by regulating miR-200c-3p/Sirt1 [75]. Collectively, lncRNA MALAT1 can regulate autophagy in IS through sponging miRNA and abolishing their effects on autophagy-related factors.

#### 3.3.3. lncRNA SNHG12

Recently, lncRNA SNHG12 was found to be significantly elevated in mouse MCAO models and OGD/R models in SH-SY5Y cells [72, 76]. An in vitro study has confirmed that overexpression of lncRNA SNHG12 could promote LC3-II and Beclin1 expression levels and the survival of SH-SY5Y cell lines after OGD/R, while downregulation of SNHG12 rescued these effects. Besides, autophagy inhibitor 3-MA can weaken the protective effect of lncRNA SNHG12 overexpression on I/R injury, suggesting that lncRNA SNHG12, as an autophagy inducer, can attenuate brain I/R injury and may be a new therapeutic target for ischemic stroke [76]. Does lncRNA SNHG12 regulate mTOR, a classical autophagy signaling pathway, or autophagy-related proteins? The mechanisms of lncRNA SNHG12 on autophagy following IS remain to be elucidated.

#### 3.3.4. lncRNA KCNQ10T1

lncRNA KCNQ1OT1 is significantly upregulated in the peripheral blood of patients with ischemic stroke. Previous studies have shown that lncRNA KCNQ1OT1 is associated with risk factors for ischemic stroke, such as diabetes and myocardial infarction [77]. A recent study substantiated that lncRNA KCNQ1OT1 silencing reduced cerebral infarction volume and alleviated neurological deficits in mouse MCAO models as well as improved cell viability of OGD/R-treated SH-SY5Y cells. Besides, rapamycin, an autophagy inducer, reversed these effects, suggesting that the downregulation of lncRNA KCNQ1OT1 protects neurons from ischemia injury by inhibiting autophagy. Further studies found that lncRNA KCNQ1OT1 may be the ceRNA of miR-200a and prevented targeting of FOXO3 and thus promoted the expression of ATG7 which participates in vesicle elongation. These experiments indicated that the knockdown of KCNQ1OT1 may inhibit the formation of autophagosomes through the miR-200a/FOXO3/ATG7 axis and increase cell viability. This finding provides a potential novel strategy for the treatment of ischemic stroke [78].

### 3.4. lncRNA-Mediated Autophagy in Epilepsy

Epilepsy (EP), as one of the most common neurological disorders, is a kind of chronic syndrome mainly caused by abnormal discharge of brain neurons [79]. It is characterized by spontaneous seizures with a high recurrence rate of 60%. Despite the availability of antiepileptic drugs (AEDs), many patients still suffer refractory seizures and unacceptable side effects [80]. Currently, the pathogenesis of epilepsy has not been well defined and the reports about the role of autophagy in epilepsy remain rare. Wu et al. showed that lncRNA MALAT1 was obviously upregulated in the hippocampus of rats with EP and the expression of LC3II/LC3I and Beclin1 also upregulated compared to the control group, indicating that EP may lead to excessive autophagy in hippocampal neurons of rats [81]. In addition, further mechanism analysis showed that lncRNA MALAT1 activated autophagy by inhibiting PI3K/AKT signaling pathway while silencing MALAT1 could prohibit autophagy in hippocampal neurons of epileptic models. These results bring up a hint that downregulated lncRNA MALAT1 can protect the hippocampal neurons from excessive autophagy through activating PI3K/AKT signaling pathway, contributing to attenuating neuron injury after EP. These findings may help to elucidate the pathophysiology of epilepsy and provide a potential therapeutic target.

### 3.5. lncRNA-Mediated Autophagy in Glioma

Glioma has been considered to be the most common type of primary brain tumor with high mortality and poor prognosis, accounting for 30% of central nervous system tumors and 80% of all malignant brain tumors [82]. The growth and metastasis of glioma rely on angiogenesis, which is one of the main reasons for treatment failure [83]. Cisplatin, a chemotherapeutic agent, binds to DNA and causes DNA damage-induced tumor cell death, which is extensively used for the treatment of glioma currently [84]. The role of autophagy in cancer varies from person to person. On the one hand, autophagy contributes to maintaining cellular homeostasis and can suppress tumor growth; on the other hand, autophagy may also facilitate proliferation and survival of tumor cells thus promoting tumor growth, invasion, and metastasis [85, 86]. In recent years, more and more studies have shown that autophagy played a key role in the development of tumors, including cell proliferation, metastasis, and chemotherapy resistance [87].

Interestingly, the latest evidence suggested that lncRNAs are involved in the development and cisplatin sensitivity of glioma through regulating autophagy. In the following paragraphs, we will discuss the regulatory lncRNAs in autophagy during glioma development and their specific mechanisms to explore a more suitable therapeutic target for treating glioma.

#### 3.5.1. lncRNA MEG3

lncRNA MEG3 is widely recognized as a tumor suppressor gene in several types of human cancers. lncRNA MEG3 was found to be markedly downregulated in glioma tissues and cell lines, which is an independent biomarker of poor prognosis in glioma [88]. It usually functioned as a ceRNA; for example, it could prevent miR-19a and miR-93 from interacting with their target mRNAs and elevate the expression levels of PTEN and PHLPP2 respectively, which inhibited PI3K/AKT/mTOR pathway and eventually suppressed the proliferation of glioma [89, 90]. Xu et al. revealed that overexpression of lncRNA MEG3 repressed cell proliferation and migration but promoted autophagy in U251 cells. Beclin1 and LC3-II/LC3-I were upregulated whereas P62 was downregulated; interestingly, these autophagy-related proteins' expressions were still unchanged after lncRNA MEG3 silencing, suggesting that autophagy in U251 cells was induced by inhibiting autophagosome degradation after overexpression of lncRNA MEG3, and inhibition of autophagy can improve cell viability. Furthermore, lncRNA MEG3 overexpression decreased the phosphorylation levels of key kinases in PI3K/AKT/mTOR pathways, indicating the inactivation of the PI3K/AKT/mTOR pathways and the upregulation of Sirt7 that is related to the deacetylation of autophagy- related genes and participated in various types of cancers [91]. However, lncRNA MEG3 or Sirt7 silencing exhibited the utter opposite effects. Collectively, lncRNA MEG3 decreased the phosphorylation levels of PI3K/AKT/mTOR pathways by enhancing Sirt7 and finally activates autophagy and improves the prognosis of glioma [92]. These results may provide novel strategies of glioma treatment, but the specific molecular mechanisms between MEG3 and Sirt7 require further investigation, and the functional role of MEG3 needs to be verified in in vivo experiments for future clinical application.

Another study showed that the expression levels of lncRNA MEG3 in U87 cells were induced by cisplatin in a time- and dose-dependent manner. Overexpression of lncRNA MEG3 enhanced the chemosensitivity of U87 cell lines to cisplatin through inhibiting cisplatin-induced autophagy, whereas knockdown of lncRNA MEG3 increased resistance of U87 cell lines to cisplatin by promoting cisplatin-induced autophagy [93]. These studies suggest that lncRNA MEG3 may be a potential target for the treatment of cisplatin-resistance glioma.

#### 3.5.2. lncRNA PVTI

A recent study found that lncRNA PVT1 was upregulated in glioma vascular endothelial cells and miR-186 was downregulated. Moreover, lncRNA PVT1 overexpression or miR-186 knockdown increased the expression levels of ATG7, Beclin-1, and LC3-II/LC3-I whereas it decreased the level of P62, contributing to cell proliferation, migration, and angiogenesis. Meanwhile, autophagy inhibitors could reverse these effects. Further mechanism analysis suggested that lncRNA PVT1 was bound to miR-186 directly and abolished its negative effects of ATG7 and Beclin-1 which is essential for autophagy initiation and the formation of a double-membrane structure. These studies are a hint that lncRNA PVT1 induces autophagy and thus promotes proliferation, migration, and angiogenesis of glioma-conditioned vascular endothelial cells through regulating miR-186-ATG7/Beclin-1 expression [94]. lncRNA PVT1 and miR-186 would provide an antiangiogenic target for gliomas.

#### 3.5.3. lncRNA MALAT1

It has been reported that lncRNA MALAT1 was highly expressed in glioma tissue and served as an indicator for poor prognosis in glioma patients; however, the regulatory mechanism of lncRNA MALAT1 in human glioma was rarely studied [95]. Recently, lncRNA MALAT1 was found to be highly expressed in glioma tissues compared with adjacent normal tissues, and its elevated expression was positively associated with the LC3-II level. In vitro experiments also showed that lncRNA Malat1 significantly promoted autophagy and proliferation of glioma cells. More importantly, the inhibition of autophagy by 3-MA alleviated MALAT1-induced glioma proliferation, suggesting that lncRNA MALAT1 could activate autophagy and eventually promote glioma proliferation. Further molecular mechanism analysis revealed that lncRNA MALAT1 could directly bind to miR-101 and prevent it from interacting with the 3′-UTR of STMN1, RAB5A, and ATG4D mRNA [96]. STMN1, RAB5A, and ATG4D were shown to be important autophagic regulators. STMN1 and RAB5A affected the fusion of autophagosomes with lysosomes, whereas ATG4D participated in autophagosome maturation [97–99]. These experimental results demonstrated that lncRNA MALAT1 promoted autophagy and proliferation of glioma cells by regulating the Malat1-miR-101-STMN1/RAB5A/ATG4D network [96]. In the latest study, lncRNA MALAT1 also acted as a miRNA sponge to regulate autophagy in glioma cells. Knockdown of lncRNA MALAT1 could depress glioma cell autophagy, migration, and invasion, whereas inhibiting miR-384 could eliminate these effects [100]. Besides, GOLM1, a downstream target of miR-384, was also identified to promote autophagy by activating protein kinase AKT [101], suggesting that lncRNA MALAT1, as a miR-384 sponge, promoted vesicle nucleation and thus enhanced glioma migration and invasion by upregulating GOLM1 [100]. This newly discovered lncRNA MALAT1/miR-384/GOLM1 axis may provide new insights into the mechanisms of glioma metastasis, and lncRNA MALAT1 may be a promising target for future glioma therapy.

#### 3.5.4. lncRNA GAS5

It has been reported that lncRNA GAS5 suppressed glioma stem cell proliferation and high expression level of lncRNA GAS5 was associated with the 2-year overall survival rate of patients with glioma [102]. Recently, a study showed that lncRNA GAS5 downregulation reduced the sensitivity of U87 cells that had high GAS5 levels to cisplatin. In contrast, lncRNA GAS5 overexpression reduced U138 cells that had a relatively low GAS5 levels resistance to cisplatin. These results suggested that lncRNA GAS5 may increase the sensitivity of glioma cells to cisplatin and play an important role in glioma chemoresistance. A Further mechanism study revealed that exposure to cisplatin could increase the expression levels of LC3II and decrease P62 levels, thus leading to excessive autophagy. In addition, upregulated lncRNA GAS5 activated mTOR signaling that was restrained by cisplatin and eventually inhibited cisplatin-induced excessive autophagy. Interestingly, blocking the mTOR pathway could also overturn the positive effect of lncRNA GAS5 upregulation on chemosensitivity to cisplatin. However, how lncRNA GAS5 regulates the mTOR signaling pathway needs to be further studied. In a word, lncRNA GAS5 suppressed cisplatin-induced excessive autophagy and thus increased cisplatin sensitivity in an mTOR-independent manner, suggesting that lncRNA GAS5 was a potential and promising target for overcoming glioma chemoresistance [103].

## 4. Conclusion and Perspectives

The above-mentioned studies have corroborated the involvement of lncRNAs in autophagy and have opened the way for further investigations into the function and mechanism of lncRNAs in neurological diseases. At present, RNA-targeted or RNA-based therapeutic approaches include antisense oligonucleotides (ASOs), RNA interference (RNAi), and ribozymes with intrinsic catalytic activities and RNA aptamers [104]. It was shown that ASOs regulated noncoding RNAs via the following two main methods. One is to synthesize antagonists to inhibit noncoding RNAs binding to their mRNA targets. The other use of ASOs is to prepare miRNA mimetics to restore levels of miRNAs that have been reduced in pathogenic conditions [105]. In 2016, two ASOs, eteplirsen and nusinersen, were approved by the FDA for the treatment of Duchenne muscular dystrophy and spinal muscular atrophy respectively, which opened a new era of ASO therapies for neurodegenerative diseases [106]. Currently, a phase 1 clinical trial of a MAPT-targeting ASO and LRRK2 ASO BIIB094 has been initiated in mild AD patients (NCT03186989) and PD patients (NCT03976349), respectively. Unlike ASO therapeutics which can be ended by terminating treatment, RNAi-based therapeutics when delivered as an shRNA can develop nonreversible efficacy, which may be beneficial but also comes with risk since it may cause a decrease in untargeted proteins [107]. A growing body of evidence points towards the promise of nanoparticles as carriers for siRNA and shRNA therapeutics for neurodegenerative disorders, including shRNAs targeting a *α*-synuclein in a mouse model of PD, siRNAs targeting BACE1 and APP in mouse CNS toward treating AD [104]. Nevertheless, these promising RNA-based therapies also face substantial challenges. Firstly, most RNA-targeted drugs cannot cross the blood brain barrier and has to be delivered to the central nervous system through intrathecal injection, an invasive delivery method that limits its use. Secondly, it is well known that lncRNA has a variety of biological functions and complex potential mechanisms; however, at present, studies on the mechanism of lncRNA are generally limited to finding relevant miRNA or binding proteins, not to mention poor conservation of lncRNA among species. Lastly, neurons possess limited ability to regenerate so that considerable undetected and potentially irreversible damage is likely to have occurred before a patient reports symptom to a physician, which requires us to find reliable biomarkers for early diagnosis and treatment. For example, in vivo and in vitro, certain lncRNA dynamically changes over ischemia time, and thus, the level of lncRNA in blood samples may reflect the pathophysiological state of the brain, which may be used as a biomarker in clinical application like myocardial markers in the future. In conclusion, this review provides an overview of lncRNAs in autophagy regulation and new insights into the underlying mechanisms and may be able to provide new ideas for studying the possible role of lncRNAs on regulating the press of neurological diseases.

## Figures and Tables

**Table 1:** lncRNAs associated with autophagy regulation in neurological diseases.

Neurological diseases	lncRNA	Main target gene	Expression	Mechanism	Function	Models	Ref.
Alzheimer's disease	17A	GABABR2	Up	17A downregulation promotes autophagy by elevating GABABR2 expression	Inducing A*β* secretion and increment of the A*β*42/40 ratio	SH-SY5Y cells treated with amyloid *β* peptide1-42	[41, 42]
	NEAT1	NEDD4L and PINK1	Up	Inhibiting PINK1-independent mitophagy by promoting NEDD4L-mediated PINK1 ubiquitination and degradation	Increasing A*β* accumulation	APP/PS1 mice	[46]
Parkinson's disease	NEAT1	PINK1	Up	Promoting autophagy through stabilizing PINK1 protein	Alleviating dopaminergic neuronal injury	MPTP mouse model and MPP+-induced SH-SY5Y	[51]
	SNHG1	miR-221/222	Up	SNHG1 downregulation increased LC3-II levels through the miR-221/222/p27/mTOR pathway	Enhancing *α*-synuclein aggregation thus leads to dopaminergic toxicity	MPTP mouse model and MPP+-induced MN9D cells	[56]
	HOTAIR	LRRK2	Up	Promoting autophagy by improving LRRK2 mRNA stability and activating ERK/MAPK pathways	Increasing the loss of striatal dopamine and promoting PD progression	MPTP mouse model and MPP+-induced SH-SY5Y	[57]
		miR-126-5p		Suppressing autophagy trough HOTAIR/miR-126-5p/RAB3IP axis	Elevating the loss of striatal dopamine and the accumulation of *α*-synuclein	MPTP rat model and MPP+-induced SH-SY5Y	[59]
	HAGLROS	miR-100	Up	Inhibiting autophagy via regulating miR-100/ATG10 axis and PI3K/AKT/mTOR pathway activation	Contributing to the development of PD	MPTP mouse model and MPP+-induced SH-SY5Y	[61]
Ischemic stroke	H19	DUSP5	Up	Promoting autophagy through regulating DUSP5-ERK1/2 axis	Aggravating cerebral ischemia-reperfusion injury	SH-SY5Y cell culture	[71]
	SNHG12	Unknown	Up	Activating beclin1-dependent autophagy	Alleviating cerebral ischemia-reperfusion injury	SH-SY5Y cell culture	[76]
	MALAT1	miR-26b	Up	Activating autophagy through upregulation of ULK2	Attenuating ischemia-reperfusion injury	BMEC culture	[74]
		miR-200c-3p		Promoting autophagy by regulating miR-200c-3p/Sirt1 axis			[75]
		miR-30a		Promoting autophagy via MALAT1/miR-30a/beclin1 axis	Aggravating ischemic injury	Mouse brain and N2a cells	[73]
	KCNQ1OT1	miR-200a	Up	Promoting the formation of autophagosomes through the mir-200a/FOXO3/ATG7 axis	Aggravating neurological impairments	Mouse brain and SH-SY5Y cell culture	[78]
Epilepsy	MALAT1	PI3K/AKT	Up	Activating autophagy by inhibiting PI3K/AKT signaling pathway	Promoting the degeneration and necrosis of hippocampal neurons	Rats with EP	[81]
Glioma	MEG3	PI3K/AKT/mTOR	Down	Activating autophagy by downregulating of the PI3K/AKT/mTOR pathways and the upregulating of Sirt7	Repressing cell proliferation and migration	U251 cell lines	[92]
		Unknown		Suppressing cisplatin-induced excessive autophagy	Increasing glioma cell sensitive to cisplatin	U87 cell lines	[93]
	PVT1	miR-186	Up	Activating autophagy through upregulation of ATG7 and Beclin1	Promoting proliferation, migration and angiogenesis	hCMEC/D3	[94]
	MALAT1	miR-101	Up	Activating autophagy through upregulation	Promoting cell proliferation	Glioma tissue from patients, U87, U118,	[96]
				STMN1, RAB5A, and ATG4D		U251, U373, and D247 cell lines	
		miR-384		Activating autophagy through upregulation GOLM1	Enhancing glioma migration and invasion	Glioma tissue from patients, SHG-44, and LN229 cells	[100]
	GAS5	mTOR	Up	Suppressing cisplatin-induced excessive autophagy in an mTOR-independent manner	Increasing glioma cell sensitive to cisplatin	U87 and u138 cell lines	[103]

## Data Availability

No data were used to support this study.
